# Sporadic Retinoblastoma and Parental Smoking and Alcohol Consumption before and after Conception: A Report from the Children’s Oncology Group

**DOI:** 10.1371/journal.pone.0151728

**Published:** 2016-03-18

**Authors:** Saeedeh Azary, Arupa Ganguly, Greta R. Bunin, Christina Lombardi, Andrew S. Park, Beate Ritz, Julia E. Heck

**Affiliations:** 1 Department of Epidemiology, UCLA Jonathan and Karin Fielding School of Public Health, Los Angeles, CA, United States of America; 2 Department of Genetics, University of Pennsylvania, Philadelphia, Pennsylvania, United States of America; 3 Division of Oncology, Center for Childhood Cancer Research, Children’s Hospital of Philadelphia, Philadelphia, PA, United States of America; National Health Research Institutes, TAIWAN

## Abstract

**Background:**

Retinoblastoma is the most frequent tumor of the eye in children and very little is known about the etiology of non-familial (sporadic) retinoblastoma. In this study we examined whether parental tobacco smoking or alcohol consumption (pre- or post-conception) contribute to the two phenotypes (bilateral or unilateral) of sporadic retinoblastoma.

**Methods:**

Two large multicenter case-control studies identified 488 cases through eye referral centers in the United States and Canada or through the Children’s Oncology Group. Controls (n = 424) were selected from among friends and relatives of cases and matched by age. Risk factor information was obtained via telephone interview. We employed multivariable logistic regression to estimate the effects of parental tobacco smoking and alcohol consumption on retinoblastoma.

**Findings:**

Maternal smoking before and during pregnancy contributed to unilateral retinoblastoma risk in the child: year before pregnancy conditional Odds Ratio (OR), 8.9; 95% confidence interval (CI) 1.5–51, and unconditional OR, 2.4; 95% CI, 1.3–4.7; month before or during pregnancy, conditional OR, 3.3; 95% CI, 0.5–20.8, and unconditional OR, 2.8; 95% CI, 1.1–7.0. No association was found for maternal or paternal alcohol consumption.

**Conclusion:**

The results of this study indicate that maternal active smoking during pregnancy may be a risk factor for sporadic retinoblastoma. Our study supports a role for tobacco exposures in embryonal tumors.

## Introduction

Retinoblastoma is a rare malignancy, usually diagnosed in early childhood, which originates in the retinal layer of the eye and results from a loss or mutation of both alleles of the *RB1* tumor suppressor gene in a retinal progenitor cell during embryonal development [1,2]. This retinal tumor causes vision loss and can become life threatening if not treated [3,4]. The 10-year survival rate is 93% in the US [5], but ranges from 40% in low income countries to 79% in upper-middle income countries [6]. When affected children have a family history of retinoblastoma, cases are considered familial (6–10% of all retinoblastoma cases), otherwise they are considered sporadic [7]. Sporadic retinoblastoma can be bilateral or unilateral. In sporadic bilateral retinoblastoma (40% of sporadic cases) the initial mutation occurs before conception in parental germ cells, and the second mutation occurs somatically after conception. In sporadic unilateral disease (60% of sporadic cases), both *RB1* allele mutations happen after conception in somatic cells of the developing retina [8,9]. The role of *RB1* mutations in retinoblastoma development is well understood, however, little is known about the etiology of these mutations and the possible role of environmental exposures [1]. It has been shown that 85% of initial germline mutations in sporadic bilateral retinoblastoma occur in the father’s allele [10], which suggests the importance of examining paternal pre-conception exposures as potential risk factors for bilateral retinoblastoma development. Because the somatic changes predisposing to unilateral disease occur in pregnancy or early life, maternal exposures during pregnancy are the most relevant potential risk factors for unilateral retinoblastoma development.

Previous studies have shown associations between parental tobacco smoke and some childhood cancers [11,12,13,14,15], however few addressed retinoblastoma. Only two studies have examined paternal smoking in relation to retinoblastoma, and results were inconsistent [16,17]. Of the four studies that examined maternal smoking, two reported increased risks for smoking during pregnancy [13,17], while a third study found a weak inverse association [16]; a separate study found a positive association with smoking prior to pregnancy [18]. However, nearly all previous studies were small in size, did not take laterality into consideration (with the exception of Bunin *et al* [17]), and included familial cases [13,16], which would be expected to attenuate associations.

Passive exposure to environmental tobacco smoke in pregnancy is additionally of concern as it can induce somatic mutations in infants born to mothers exposed during pregnancy [19]. Currently, there are no published studies that have investigated passive exposure to environmental tobacco smoke in pregnancy and retinoblastoma development. In addition, there have been only two studies that reported on maternal or paternal alcohol drinking during or prior to pregnancy and these found no association, although sample sizes were small and alcohol consumption was quite low in the Mexican study, making it difficult to draw conclusions [17,20].

In this multi-center case control study, we investigate the associations between parental smoking and drinking before and during pregnancy and retinoblastoma development in the child. Our study is the first to investigate bilateral and unilateral cases separately and assess exposures during two different periods of relevance i.e. before and after conception, focusing on pre-conception exposures among fathers and bilateral retinoblastoma, and maternal pregnancy exposures and unilateral retinoblastoma.

## Materials and Methods

### Sample/Participants

Data were derived from two large case-control studies of retinoblastoma [21,22]. In the first study, cases (n = 206) were diagnosed with sporadic bilateral retinoblastoma from January 1998 to May 2006 at nine large referral centers for retinoblastoma [Wills Eye Hospital (Philadelphia), Northwestern University Medical Center (Chicago), New York Hospital/Cornell University Medical Center (New York), Children’s Hospital of Los Angeles, The Hospital for Sick Children (Toronto), Children’s Hospital and Regional Medical Center (Seattle), St. Jude Children’s Research Hospital (Memphis), the University of Illinois at Chicago, and the Children's Hospital of Philadelphia]. Institutional review boards (IRBs) at each of these institutions approved the study. The second study included cases (n = 282) who were diagnosed with sporadic retinoblastoma (both unilateral and bilateral disease) and ascertained through Wills Eye Hospital in Philadelphia or the Children’s Oncology Group (COG) between June 2006 and June 2011. The second study was approved by the IRBs of the University of Pennsylvania, Children’s Hospital of Philadelphia, and Wills Eye Hospital. In addition, all institutions that enrolled patients obtained IRB approval from their own IRB or through the Central IRB. Participants included children from COG institutions across North America. In the first study 206 cases (all bilateral cases) and 269 controls were enrolled, and the second study enrolled 282 cases (187 unilateral and 95 bilateral cases) and 155 controls. The present analysis was approved by the Human Subjects Protection Board of the University of California, Los Angeles.

Cases were eligible for the studies if the child’s physician gave permission to contact the patient’s family, the biologic father and/or mother were available, the family had a telephone, the family resided in the US or Canada and spoke English or Spanish. Once the physician obtained consent, participants’ information was shared with researchers for follow-up with letters and interviews. A letter was sent to the parents explaining the study and inviting them to participate. In order to determine if disease was familial or sporadic, we obtained buccal swabs or saliva from the child and both parents to investigate cells for the presence of *RB1* mutations; enrollment in the studies was limited to sporadic cases. Written informed consent was obtained when participants provided biospecimens. In both studies, because interviews took place via telephone, we sought a separate verbal informed consent from participants for the interview. Consent was documented by the interviewer on the questionnaire. Consent procedures were approved by all IRBs.

Case families were asked to suggest controls for the study from among their relatives or friends who had a child in the same age group as the case (0–1, 2–4, 5–9, 10–14 years old). For bilateral cases we aimed to recruit control children whose father was not a biological relative of the case’s father, and for unilateral cases we aimed to recruit a control child whose mother was not a biological relative of the case’s mother; as such, “ideal controls” were considered both age-matched and non-biological relatives. The rationale for this, as explained above, is the predominance of *de novo* germline mutations arising from the paternal allele in bilateral retinoblastoma, whereas the somatic changes which cause unilateral retinoblastoma arise during pregnancy. Some case families were unable or reluctant to suggest any friend or relative as a control and in these instances we do not have matched controls, while others were able to nominate 2 or 3 friends or relatives as controls. Controls were recruited in the same manner as cases, via a letter inviting them to participate followed by a call to schedule an interview. In some instances, we were not able to recruit an “ideal” control child, thus we enrolled some controls (7.8% of the control sample) that were not age-matched or biological relatives, but were willing to participate. Some control interviews were completed when the corresponding case parent never completed the interview; the present analysis also includes these 92 ‘unmatched’ controls with their responses contributing to unconditional analyses. More details describing recruitment procedures are provided in the supplement (S1 Document) and in supplementary figures (S1 and S2 Figs).

### Parental smoking

In structured telephone interviews with one or both parents of participants, we collected data on demographics, tobacco and alcohol use, and other exposures, including time-specific information on substance use (lifetime, the year prior to pregnancy, during pregnancy, and trimester-specific). We collected information on cigarette smoking only and did not include other types of tobacco products. We defined ever smoking as having ever smoked more than 100 cigarettes in the participant’s lifetime. We calculated pack-years of tobacco smoking from the average number of cigarettes smoked per day and the duration of use (classified as 0, >0–5, >5–10, >10 pack-years). Among the fathers in our study who reported smoking, the mean pack-years of smoking was 9.3 (range 0.05–61.3) and the median was 6. Among all smoking fathers, 41% had smoked less than 5 pack-years and 30% had smoked more than 10 pack-years. Cigarettes smoked per day were grouped into three categories (0, 1–9, 10+ cigarettes per day). For smoking mothers, the mean number of cigarettes smoked per day was 8.7 (range 1 to 40) and the median was 6. For smoking fathers, the mean number of cigarettes per day was 12.8 (range 1 to 60) and the median was 10.

### Parental alcohol use

Parents also reported how many drinks they had (per day/week/month) and how often they had more than 6 drinks per occasion (binge drinking), with a drink defined to participants as a beer, a glass of wine, or a shot of liquor. Among alcohol-drinking fathers, the mean number of drinks per week was 6.5 (range from 1 per year to 90 per week) and the median was 3 per week. For alcohol-drinking mothers, the mean number of drinks per week was 2.6 (range from 1 per year to 42 per week) and the median was 1 per week. Although both studies ascertained general smoking and drinking information, only the second study collected the mother’s smoking and drinking during pregnancy and within each trimester.

### Statistical analysis

We estimated associations using both conditional and unconditional logistic regression models, breaking the matches since we did not have a matched control for every case. Thus, we estimated Mantel-Haenszel conditional logistic regression for matched pairs, reflecting the original study design, and we also used unconditional logistic regression models which allowed us to use the information from all available cases and controls. There were 95 matched unilateral retinoblastoma cases with 95 matched controls, and 188 matched bilateral retinoblastoma cases with 298 matched controls. The number of matched controls differed due to differing matching ratios i.e. among bilateral cases, 107 were only matched to one control, 52 to two controls, and 29 to three controls. In unconditional logistic regression models we adjusted for the matching variable, age. In analyses of bilateral retinoblastoma we adjusted for father’s race (White non-Hispanic, African American non-Hispanic, Hispanic, other), father’s educational attainment (i.e. less than high school, high school graduate, some college or other training, and college graduate or more), household income (i.e. <$35,000, $35,000 to 50,000, $50,000 to 75,000, >$75,000), and the father’s age at child’s birth (<25, 25–29, 30–34, 35–39, 40+). When the exposure of interest was smoking, we additionally adjusted for father’s drinking, and vice versa. In analyses of unilateral retinoblastoma we adjusted for mother’s race, educational attainment, household income, and mother’s age at child’s birth. We mutually adjusted for the spouse’s smoking or drinking; ie. when investigating the association between paternal smoking and retinoblastoma, we adjusted for maternal smoking. Other covariates including marital status, interview by proxy, and child’s gender did not change estimates by more than 10% and were not included in final models.

Because the second allele in bilateral retinoblastoma is lost during the pregnancy, we additionally examined if maternal smoking in pregnancy was related to bilateral retinoblastoma. In order to ascertain transgenerational effects on the mother’s germ cells, we additionally examined the association between retinoblastoma risk in the child and the grandmother’s smoking while she was pregnant with the mother of the index child.

Because disclosure of cigarette smoking by pregnant mothers to interviewers differs by race/ethnicity [23,24], we conducted sensitivity analyses to examine the association between smoking and retinoblastoma within white non-Hispanic mothers only.

We also conducted sensitivity analyses examining results when excluding participants who were not “ideal” matches (7.8% of controls), as defined above. All analyses were conducted using SAS (Cary, NC).

## Results

The demographics of study participants in both studies were similar with regards to parental race/ethnicity and age. The second study included slightly more fathers (56% vs. 48%) and mothers (59% vs. 50%) with a college degree or more. The demographics of matched cases and controls and conditional regression results are shown in Tables 1–5 while unmatched cases and controls and unconditional regression results are presented in supplemental tables.

**Table 1:** Demographic characteristics of fathers of the bilateral cases and matched controls.

	Matched ^a^	
	Controls	Bilateral cases
Characteristics	(N = 298)	(N = 188)
	N (%)	N (%)
**Father's race**		
White non-Hispanic	236 (79.5)	138 (73.4)
African American non-Hispanic	21 (7.1)	20 (11.6)
Hispanic	26 (8.8)	19 (10.1)
Other	14 (4.7)	11 (5.9)
Missing	1	0
**Father's educational attainment**		
<High school	14 (4.7)	98 (4.3)
High school graduate	52 (17.5)	45 (25.1)
Some college or other training	60 (20.1)	40 (21.4)
College graduate or more	172 (57.7)	94 (50.3)
Missing	0	1
**Father's age at child’s birth**		
<25	25 (8.6)	16 (8.6)
25–29	51 (17.5)	31 (16.7)
30–34	112 (38.4)	61 (32.8)
35–39	72 (24.7)	55 (29.6)
40+	32 (11)	23 (12.4)
Missing	6	2
**Total household income**		
Less than $ 35,000	48 (18.2)	30 (17.3)
35,000–50,000	43 (16.3)	35 (20.2)
50,000–75,000	64 (24.2)	38 (22.0)
More than $ 75,000	105 (39.8)	67 (38.7)
Refused	3 (1.1)	1 (0.6)
Do not know	1 (0.4)	2 (1.2)
Missing	34	15

^a^ There were 107 cases with one matched control, 52 cases with two matched controls, and 29 cases with three matched controls.

**Table 2:** Demographic characteristics of mothers of the unilateral cases and matched controls.

	Matched case-control pairs	
	Controls	Unilateral cases
Characteristics	(N = 95)	(N = 95)
	N (%)	N (%)
**Mother's race**		
White non-Hispanic	74 (77.9)	65 (68.4)
African American non-Hispanic	2(2.1)	3 (3.2)
Hispanic	11 (11.6)	19 (20.0)
Other	8 (8.4)	8 (8.4)
Missing	0	0
**Mother's educational attainment**		
<High school	3 (3.2)	5 (5.3)
High school graduate	14 (14.7)	13 (13.7)
Some college or other training	17 (17.9)	15 (15.8)
College graduate or more	61 (64.2)	62 (65.3)
Missing	0	0
**Mother's age at child’s birth**		
<25	14 (14.7)	9 (9.5)
25–29	30 (31.6)	39 (41.1)
30–34	37 (39.0)	32 (33.7)
35–39	12 (12.6)	11 (11.6)
40+	2 (2.1)	4 (4.2)
Missing	0	0
**Total household income**		
Less than $35,000	22 (23.2)	23 (24.2)
35,000–50,000	9 (9.5)	13 (13.7)
50,000–75,000	19 (20.0)	20 (21.1)
More than $ 75,000	41 (43.2)	36 (37.9)
Refused	1 (1.1)	1 (1.1)
Do not know	3 (3.2)	2 (2.1)
Missing	0	0

**Table 3:** Paternal smoking and alcohol consumption and bilateral retinoblastoma (Conditional logistic regression).

	Controls	Bilateral cases		
	(N = 298)	(N = 188)	Crude	Adjusted
	N (%)	N (%)	OR	OR (95% CI)^a^
**Father ever smoked, lifetime**				
No	187 (62.7)	117 (62.2)		
Yes	111 (37.3)	71 (37.8)	1.0	1.0 (0.6, 1.6)
Missing	0	0		
**Father's lifetime smoking (pack-years)**				
0	187 (65.9)	117 (66.5)	1.0	
>0 to 5	49 (17.3)	25 (14.2)	0.8	0.8 (0.4, 1.5)
>5 to 10	27 (9.5)	13 (7.4)	0.8	1.0 (0.4, 2.4)
>10	21 (7.4)	21 (11.9)	1.6	1.2 (0.5, 2.9)
Missing	4	12		
**Father smoked in the year before pregnancy**				
Never smoked, lifetime	187 (62.8)	117 (62.2)	1.0	
Ever smoker, did not smoke in year before pregnancy	46 (15.4)	24 (12.8)	0.8	0.9 (0.5, 1.8)
Smoked in year before pregnancy	65 (21.8)	47 (25.0)	1.1	1.0 (0.5, 1.8)
Missing	0	0		
**Father's number of cigarettes per day, year before pregnancy**				
0	233 (79.0)	141 (75.4)	1.0	
1–9	26 (8.8)	18 (9.6)	1.1	0.6 (0.2, 1.8)
10+	36 (12.2)	28 (15.0)	1.3	1.4 (0.6, 3.2)
Missing	3	1		
**Father drinking alcohol, year before pregnancy**				
0 drink	59 (19.8)	37 (19.7)	1.0	
<1 drink per week	51 (17.1)	29 (15.4)	0.8	0.6 (0.2, 1.2)
1–7 drinks per week	127 (42.6)	83 (44.2)	0.9	0.6 (0.3, 1.3)
1+ drinks per day	61 (20.5)	39 (20.7)	0.8	0.6 (0.2, 1.3)
Missing	0	0		
**Father's drinking ≥ 6 drinks per occasion in the year before pregnancy (binge drinking)**				
No	189 (63.4)	121 (64.4)	1.0	
Yes	109 (36.6)	67 (35.6)	0.8	0.7 (0.4, 1.3)
Missing				
**Father's drinking ≥ 6 drinks per occasion in the year before pregnancy (binge drinking)**				
Never	189 (63.4)	121 (64.4)	1.0	
< Once per month	54 (18.1)	44 (23.4)	1.0	1.0 (0.5, 2.0)
Monthly	31 (10.4)	14 (7.5)	0.6	0.7 (0.3, 1.5)
Daily or Weekly	24 (8.1)	9 (4.8)	0.5	0.4 (0.1, 1.2)
Missing	0	0		

^a^ Smoking and drinking analyses adjusted for father's race, father's educational attainment, father's household income, and father's age at child's birth. In addition, smoking analyses adjusted for father's drinking in the year before pregnancy, and mother's smoking. Drinking analyses additionally adjusted for father's smoking in the year before pregnancy, and mother's drinking.

**Table 4:** Maternal smoking and drinking consumption and unilateral retinoblastoma (Conditional logistic regression).

	Controls	Unilateral cases		
	(N = 95)	(N = 95)	Crude	Adjusted
	N (%)	N (%)	OR	OR (95% CI)^a^
**Mother ever smoked, lifetime**				
No	69 (72.7)	56 (58.9)	1.0	
Yes	26 (27.3)	39 (41.1)	2.2	3.7 (1.1, 12.2)
Missing	0	0		
**Mother smoked in the year before pregnancy**				
Never smoked, lifetime	69 (72.6)	56 (59.0)	1.0	
Ever smoker, did not smoke in year before pregnancy	10 (10.5)	21 (19.0)	1.8	2.0 (0.5, 8.4)
Smoked in year before pregnancy	16 (16.8)	18 (22.1)	2.9	8.9 (1.5, 51)
Missing	0	0		
**Mother's cigarettes per day, year before pregnancy**				
0	85 (91.4)	77 (83.7)	1.0	
1–9	2 (2.2)	6 (6.5)	3.0	15 (0.9, 239)
10+	6 (6.5)	9 (9.8)	4.0	7.6 (0.3, 178)
Missing	2	3		
**Mother drinking alcohol, year before pregnancy**				
0	14 (14.9)	19 (20.0)	1.0	
<1 drink per week	41 (43.6)	36 (37.9)	0.6	0.6 (0.2, 2.1)
1–7 drinks per week	34 (36.2)	32 (33.7)	0.6	0.5 (0.1, 2.5)
1+ drinks per day	5 (5.3)	8 (8.4)	1.3	0.5 (0.1, 4.1)
Missing	1	0		
**Mother's drinking ≥ 6 drinks per occasion, year before pregnancy**				
No	76 (80.0)	70 (73.7)	1.0	
Yes	19 (20.0)	25 (26.3)	1.5	0.8 (0.3, 2.2)
Missing	0	0		
**Mother's smoking in the month before or during pregnancy**				
Never smoked, lifetime	67 (72.0)	56 (59.6)	1.0	
No	16 (17.2)	23 (24.5)	1.8	1.8 (0.5, 6.8)
Yes	10 (10.8)	15 (16.0)	2.2	3.3 (0.5, 20.8)
Missing	2	1		
**Mother's smoking in the first trimester**				
No	89 (95.7)	87 (92.5)	1.0	
Yes	4 (4.3)	7 (7.5)	2.0	5.1 (0.4, 72.8)
Missing	2	0		

^a^ Smoking and drinking analyses adjusted for the matching variable (child age at interview), mother's race, mother's educational attainment, household income, and mother's age at child's birth. In addition, smoking analyses are adjusted for mother's drinking in the year before pregnancy, and for father's smoking; drinking analyses are adjusted for the mother's smoking in the year before pregnancy and father's drinking.

**Table 5:** Environmental tobacco exposure of mother during pregnancy and unilateral retinoblastoma (Conditional logistic regression analysis).

	Controls	Unilateral cases	Conditional	
				Adjusted
	N (%)	N (%)	OR	OR (95% CI)^a^
***Mother never smoked*, *father and mother lived together***	64	52		
** Father ever smoked, lifetime**				
No	47 (78.3)	33 (67.4)	1.0	
Yes	13 (21.7)	16 (32.7)	1.4	1.4 (0.1, 16.8)
Missing	4	3		
** Father's lifetime smoking (pack-years)**				
0	47 (81.0)	33 (68.8)	1.0	
>0 to 5	7 (12.1)	12 (25.0)	3.0	1.4 (0.1, 16.1)
>5 to 10	3 (5.2)	2 (4.2)	1.0	NE
>10	1 (1.7)	1 (2.1)	NE	NE
Missing	6	4		
** Father smoked in the year before pregnancy**				
Never smoked, lifetime	47 (78.3)	33 (67.4)	1.0	
Ever smoker but did not smoke in the year before	7 (11.7)	8 (16.3)	1.2	0.4 (0.0, 13.0)
Smoked in the year before pregnancy	6 (10.0)	8 (16.3)	2.1	3.9 (0.1, 108.5)
Missing	4	3		
***Mother and father lived together***	87	88		
Both father and mother are never smoker, lifetime	47 (58.0)	33 (40.0)	1.0	
Father ever smoker and mother never smoker, lifetime	13 (16.1)	16 (19.5)	1.7	2.0 (0.4, 9.0)
Father never smoker and mother ever smoker, lifetime	10 (12.4)	19 (23.2)	2.6	3.3 (0.8, 13.4)
Both father and mother ever smoked, lifetime	11 (13.6)	14 (17.1)	1.8	2.7 (0.7, 11.3)
Missing	6	6		

^a^ Adjusted for mother's race, mother's educational attainment, household income, mother's age at child's birth, mother’s alcohol drinking in the year before pregnancy.

NE = Not Estimable

Comparing families with a matched control to families for which no matching control was identified, fathers of bilateral cases with matched controls (n = 188) included more White non-Hispanics than fathers of bilateral cases without matched controls (n = 106), and these fathers also had higher education and higher income than fathers of unmatched bilateral cases, but there was no difference in fathers’ age at child’s birth. There was no difference between matched controls (n = 298) and unmatched controls (n = 92) with regards to paternal race, paternal educational attainment, fathers’ age at child’s birth, and fathers’ total household income.

Table 1 presents the paternal characteristics of bilateral retinoblastoma cases and their matched controls. Control fathers had slightly higher educational attainment.

Table 2 summarizes the maternal characteristics of unilateral retinoblastoma cases and controls. Maternal demographics for unilateral cases and their matched controls were similar. Characteristics of unmatched cases and controls are shown in S1 and S2 Tables.

Mothers of unilateral cases with a matched control (n = 95) included slightly more White non-Hispanics, while mothers of unilateral cases without a matched control (n = 90) were more often of African American and Hispanic ancestry. The mothers of unilateral cases with a matched control had higher educational attainment and higher total household income than mothers of cases without a matched control. A majority of mothers of unmatched unilateral cases were less than 25 years of age at the child’s birth.

There were no differences between the matched controls of unilateral cases (n = 95) and all unmatched controls (n = 314) with regards to maternal race, maternal educational attainment, maternal age and total household income.

Associations between bilateral retinoblastoma and paternal smoking or alcohol drinking are shown in Table 3 and S3 Table. No association was observed between paternal ever smoking and bilateral retinoblastoma development (conditional OR, 1.0; 95% CI, 0.6–1.6 and unconditional OR, 1.1; 95% CI, 0.8–1.7). We observed differences between conditional and unconditional analyses for paternal exposures: in conditional analyses paternal smoking of more than 10 pack-years was not associated with retinoblastoma development in the child (OR, 1.2; 95% CI, 0.5–2.9). However, in the unmatched analysis, paternal smoking of more than 10 pack-years was associated with an increased odds of bilateral retinoblastoma in the child (OR, 1.9; 95% CI, 1.0–3.8). Paternal smoking of more than 10 cigarettes per day in the year before pregnancy showed a positive association with bilateral retinoblastoma in unconditional analyses (OR, 1.7; 95% CI, 0.9–3.1), and increased odds, with confidence intervals including the null value, in conditional analyses (OR, 1.4; 95% CI, 0.6–3.2). Regarding paternal alcohol consumption in the year before pregnancy, there was no evidence for a dose-response gradient and a negative association was found in the matched analysis (OR, 0.6; 95% CI, 0.2–1.3). No association was observed for paternal binge drinking in the year before pregnancy.

The results for maternal smoking were similar in both matched and unmatched analyses. With respect to maternal smoking, 41% of mothers of unilateral cases and 27% of mothers of controls were ever smokers, and the risk of unilateral retinoblastoma was increased in ever smokers (OR, 3.7; 95% CI 1.1, 12.2 in the conditional analysis) (Table 4), and it was 70% higher in ever smokers in the unconditional analysis (OR, 1.7; 95% CI, 1.0–2.8) (S4 Table). Tobacco smoking by the mother in the year before pregnancy increased the risk of unilateral retinoblastoma in the child (conditional OR, 8.9; 95% CI, 1.5–51, and unconditional OR, 2.4; 95% CI, 1.3–4.7). In the conditional analysis, maternal smoking in the month before or during pregnancy and in the first trimester increased the risk of retinoblastoma development in the child, but confidence intervals were very wide (OR, 3.3; 95% CI, 0.5–20.8, and OR, 5.1; 95% CI, 0.4–72.8, respectively). With regards to maternal smoking in the month before pregnancy or during pregnancy in the unconditional analysis, we similarly observed an elevated risk for unilateral retinoblastoma (OR, 2.8; 95% CI, 1.1–7.0) with the strongest effect in the first trimester (OR, 3.7; 95% CI, 1.2–11.6) (S4 Table). Results of conditional analyses for maternal smoking and drinking in each trimester, and unilateral retinoblastoma development in the child are shown in S6 Table.

Sensitivity analyses restricting to white non-Hispanic mothers suggested that the risk was highest among those who smoked in the month before or during pregnancy [OR, 5.3; 95% CI, 1.6–17.9, with 13 (12%) controls and 28 (27%) cases reporting smoking] and the risk among those who smoked in the year before pregnancy was also elevated [OR, 3.3; 95% CI, 1.5–7.4, with 49 (15.3%) controls and 33 (40.2%) cases]. Effect estimates for ever cigarette smoking and unilateral retinoblastoma were almost identical in white non-Hispanic mothers and all mothers (OR, 1.9; 95% CI, 1.0–3.4 and OR, 1.7; 95% CI, 1.0–2.8, respectively).

Maternal alcohol consumption either in the year or month before or during pregnancy did not show any association with unilateral retinoblastoma. In addition, no association was found for the father’s smoking or drinking before conception for unilateral retinoblastoma, and no relation was found for maternal smoking and drinking before conception with bilateral retinoblastoma development (data not shown).

Among maternal grandmothers, 79 (20.4%) of control grandmothers, 32 (17.3%) of unilateral case grandmothers, and 53 (18.5%) of bilateral case grandmothers were smokers during their pregnancy with the mother of the index child. We did not observe any association between the grandmother’s smoking during her pregnancy and unilateral retinoblastoma risk in the grandchild (OR, 1.1; 95% CI, 0.5–2.1) with a slightly increased risk estimate for bilateral retinoblastoma development, but confidence intervals crossed the null (OR, 1.3; 95% CI, 0.5–3.1).

Environmental tobacco smoke exposure of the mother increased the child’s risk of unilateral retinoblastoma in unconditional analyses, however the point estimate dropped in conditional analyses (conditional OR, 1.4; 95% CI, 0.1–16.8, and unconditional OR, 1.9; 95% CI, 1.0–3.6) and our data did not allow us to assess a dose-response relationship due to the small number of heavy smokers (Table 5 and S5 Table).

In analyses of maternal smoking and bilateral retinoblastoma, unconditional logistic regression results showed elevated risks with wide confidence intervals for maternal smoking in the month before or during pregnancy (OR = 1.7, 95% CI 0.6, 5.4), and risk was more elevated for smoking in the first trimester (OR = 3.7, 95% CI 1.0, 14.0). However we were not able to confirm these results in conditional regression analyses, because results could not be estimated because of the small number of matched mothers who smoked.

In sensitivity analyses, we examined the results after exclusion of parents for whom the interview was conducted by proxy and found that our results did not change (data not shown). In sensitivity analyses including only ideal controls, tobacco smoking by the mother in the year before pregnancy and the month before or during pregnancy also increased the risk of unilateral retinoblastoma in the child (OR, 4.3; 95% CI, 1.7–11.6 and OR, 3.2; 95% CI, 1.2–8.9, respectively), and we observed the strongest effect size in the first trimester (OR, 5.2; 95% CI, 1.3–20.5).The results of maternal smoking were similar among the matched and unmatched analyses (data not shown). Examining only ideal controls, paternal smoking did not show any association with bilateral retinoblastoma in the child in the matched and unmatched analyses (data not shown). However, paternal alcohol drinking of more than one drink per day in the year before pregnancy increased the risk of bilateral retinoblastoma in the child (OR, 3.4; 95% CI, 0.9–13.6).

## Discussion

Our multicenter investigation included a large number of cases for a study of embryonal tumors and differs from previous studies in that it was restricted to sporadic cases only. We had sufficient sample size to stratify by laterality, allowing us to investigate parental exposures most relevant for bilateral *vs*. unilateral disease and compare differences in timing and dose of parental exposures to tobacco or alcohol (pre- or post-conception exposures). In previous studies the amount of smoking, particularly heavier smoking, was not evaluated [13,16,17,18].

Tobacco smoke has a mutagenic effect which can cause mutations in paternal germ cells before conception; tobacco metabolites can also pass through the placenta leading to mutations in fetal cells [11–15]. Animal models showed that tobacco exposure causes spermatogonial stem cell mutations [25]. Aneuploidy, DNA adducts, strand breaks, oxidative damage, and DNA mutations are increased in the sperm of smokers [26,27]. In mice, exposure to tobacco smoke induces germ line mutations in Expanded Simple Tandem Repeats (ESTR) that can be passed to offspring and cause a permanent modification in their genome [25].

We found that paternal tobacco smoking of more than 10 pack-years and of more than 10 cigarettes per day in the year before pregnancy increased the risk of bilateral retinoblastoma in the child in unmatched analyses, however in matched analyses there were no associations, which suggests the finding may be due to underlying differences between cases and controls rather than a true association. Nonetheless, 85% of new germline mutations in retinoblastoma happen in paternal alleles before conception. Only 15% of new germline mutations originate from maternal oocytes, and it has been shown that oocytes are less sensitive to mutations than sperm [22].

The eye develops from neural ectodermal cells in the anterior region of the neural plate [28]. The optic vesicle, the source of retina development, originates in day 24 of gestation and in day 35 of gestation, the optic stalk develops and creates the external retinal layer. Although the majority of eye development happens between week 4 and 8–9 of gestation, eye development continues into postnatal life. For example, the fovea centralis of the retina, which is the central part of the retina responsible for sharp vision, does not differentiate until 4 months after birth, and other parts of the eye such as the cones, lenses, and dilator papillae muscles continue to develop in early life [29].

We observed increased risks for unilateral disease with maternal smoking during pregnancy, with the strongest effect estimates for smoking found during the first trimester of pregnancy. This is consistent with the observation that retina development is initiated between week 4 and 8 of gestation continuing through the first 6 months after birth [30,31]. Prior studies have shown that children of women who smoke in pregnancy have more Hypoxanthine-guanine Phosphoribosyl Transferase (HPRT) mutations, translocations, and DNA strand breaks [26,27]. Since it is possible that mothers who smoked prior to pregnancy continue to smoke during pregnancy, it may be that the elevated risk we observed with maternal tobacco smoking in the year before pregnancy reflects the risk from smoking during pregnancy. For instance, we observed that 60% of case mothers who were smokers in the year before pregnancy also smoked in the first trimester, and this percent may be underreported to study interviewers if case mothers felt guilty for having smoked. If case mothers systematically reported less smoking and alcohol drinking, any bias in our estimates would have been towards the null. We do not expect confounding by birth weight because in our study, as in other studies [8], retinoblastoma cases and controls had similar birth weight distributions.

Similar to previous published studies [17,20], we did not observe any association for maternal alcohol drinking in pregnancy and unilateral retinoblastoma. However, we observed a positive association in unmatched analyses for father’s daily alcohol consumption prior to conception and bilateral retinoblastoma even after adjustment for smoking. However, this association did not remain when we performed our matched analysis. Alcohol is an established carcinogen and can induce mutations in paternal sperm DNA before conception and in the child’s cells after conception [11,32,33]. Acetaldehyde, the first metabolite of ethanol, induces chromosomal aberrations, sister-chromatid exchanges and cross-links between DNA strands [11]. Formaldehyde, which is also a metabolite of methanol, is carcinogenic and reduces cellular RNA synthesis, which is a mechanism of carcinogenicity [11].

In our study, there were considerable differences between cases and controls by race/ethnicity and socioeconomic status (SES), and this was particularly a concern in unmatched analyses, where demographic differences were more stark between case and control groups. While we attempted to account for demographic differences by adjusting for parental race, age, educational attainment and household income, the possibility of residual confounding by SES remains a concern. Although high SES is generally related to less smoking and drinking [34,35], patterns in pregnancy differ: drinking tends to be higher among high SES women, while smoking tends to be higher in low SES women [36]. Because our controls had higher SES, our estimates would have been overestimated in smoking analyses and underestimated in drinking analyses. In a separate analysis of this data, our group utilized propensity scores to account for the case/control differences and we did not observe substantially different results [37].

In 2011, cigarette smoking prevalence in the United States was 19–26% among males of reproductive age, 14.3–20.9% among females of childbearing age [38], and 14% among pregnant women in 1996 [39]. In our study 22% of control fathers and 14.9% of control mothers were smokers in the year before pregnancy, suggesting that control smoking rates in our study approximated rates in the general population. Among our control mothers who had smoked during the year before pregnancy, 64% quit during the first trimester and 74% quit by the second and third trimester. In contrast, fewer case mothers who smoked in the year before pregnancy reported quitting in the first (40–45%) or second and third trimesters (50–68%). It should be noted, however, that maternal smoking during pregnancy is difficult to assess with accuracy because some mothers go through cycles of attempting to cut down or quit, with relapses followed by new attempts to quit [40].

One of the limitations of our study is the potential for misclassification of exposure. Previous studies which verified smoking status via serum cotinine assay showed that some women are reluctant to disclose their smoking status to interviewers, and when they report smoking, they commonly underreport the number of cigarettes smoked [41].This underreporting in pregnancy varies by race, with Mexican, other Hispanic, and African-American women less likely to accurately report smoking during pregnancy [23,24]. Because our sensitivity analyses showed higher effect estimates when the population was restricted to non-Hispanic Whites only, this may suggest possible underreporting among minority participants. It is not known whether underreporting would be differential by case status. The use of friend controls who may have had similar lifestyle characteristics as cases could have led to overmatching and reduced statistical power [42]. Due to overmatching, controls may be more similar to cases than the base population with respect to the exposure and bias findings towards the null. An earlier report by our group investigated the possibility of overmatching due to this study design and showed evidence of some overmatching on demographic characteristics, but little overmatching on smoking [42].

Our findings support that maternal active smoking during pregnancy is a risk factor for unilateral retinoblastoma. We did not find any association for maternal alcohol drinking in pregnancy and unilateral retinoblastoma nor any risk increase for paternal alcohol drinking before pregnancy and bilateral retinoblastoma. These results affirm earlier conclusions of increased risks of embryonal tumors with parental smoking [14]. Our findings provide another reason to educate prospective parents about the health and reproductive hazards of smoking.

## Supporting Information

S1 Document Participant recruitment. (DOCX)

S1 Fig Recruitment of case and control parents for a study of sporadic bilateral retinoblastoma (first study). (PDF)

S2 Fig Recruitment of case and control parents for a study of sporadic bilateral and unilateral retinoblastoma (second study). (PDF)

S1 Table Demographic characteristics of fathers in unmatched analyses. (PDF)

S2 Table Demographic characteristics of mothers in unmatched analyses. (PDF)

S3 Table Paternal smoking and alcohol consumption and bilateral retinoblastoma (unconditional logistic regression analysis results). (PDF)

S4 Table Maternal smoking and drinking consumption and unilateral retinoblastoma (unconditional logistic regression analysis results). (PDF)

S5 Table Second hand tobacco exposure of mother during pregnancy and unilateral retinoblastoma (unconditional logistic regression analysis results). (PDF)

S6 Table Maternal smoking and drinking and unilateral retinoblastoma, conditional logistic regression analysis results (Table 4 continued). (PDF)

## Abbreviations

OR: Odds Ratio
CI: Confidence Interval
ESTR: Expanded Simple Tandem Repeats
HPRT: Hypoxanthine-guanine Phosphor Ribose Transferase
SES: Socioeconomic Status
