# Supplementary material for: Sporadic Retinoblastoma and Parental Smoking and Alcohol Consumption before and after Conception: A Report from the Children’s Oncology Group
Source: PLoS One. 2016 Mar 18;11(3):e0151728. doi: 10.1371/journal.pone.0151728 (PMC4798297; doi:10.1371/journal.pone.0151728)
Supplement: S1 Document — (DOCX) [file pone.0151728.s001.docx]

## Participant recruitment:

In the first study, participating institutions identified 236 patients, with 30 excluded for the following reasons: a biological parent was not available because the child was in foster care or was adopted (n=2), neither parent spoke English or Spanish (n=5), inability to locate the family (n=1), physician requested no contact (n=2), parents refused (n=9), mutation testing showed an inherited RB1 mutation or mosaicism (n=11). Of the remaining 206 patients, the case’s mother (n=204) and/or father (n=203) were interviewed for the study.

Of the 374 controls who were selected in the first study, mothers of 263 (70%) and fathers of 247 (66%) completed interviews. For five controls, only the father was interviewed, and for 21 cases, only the mother was interviewed. The remaining families were excluded for the following reasons: refusal to participate (n=72), not interviewed by the end of the study (n=22), ineligible (n=12).

In the second study, participating institutions identified 130 bilateral retinoblastoma cases of which 95 mothers were interviewed. Families were excluded (n=35) for the following reasons: mutation testing showed an inherited RB1 mutation or mosaicism (n=8), refused the interview (n=7), refused the study (n=5), inability to locate (n=1), not from US or Canada (n=1), not interviewed before the end of study (n=13). Institutions also identified 242 unilateral retinoblastoma cases of which 185 mothers of unilateral cases were interviewed. Others were excluded (n=57) for the following reasons: not interviewed before the study end (n=27), refused the interview (n=15), could not be located (n=6), mutation testing showed an inherited RB1 mutation or mosaicism (n=5), refused the study (n=2), not from US or Canada (n=1), biological parent not available due to foster care or adoption (n=1).

In the second study, from the 218 identified controls introduced by cases, 147 control mothers were interviewed, and the remaining were excluded for the following reasons: refused the study (n=34), not interviewed before study end (n=21), only father interviewed (n=8), mother did not respond (n=6), or they were ineligible (n=2).

In total, interviews were completed with 461 case fathers (294 bilateral and 167 unilateral cases), 390 control fathers, 484 case mothers (299 bilateral and 185 unilateral cases) and 410 control mothers. Interviews lasted on average an hour. Some maternal (2.4%) and paternal (16.8%) interviews were conducted via a proxy respondent, typically the other parent.
