# Supplementary material for: Sporadic Retinoblastoma and Parental Smoking and Alcohol Consumption before and after Conception: A Report from the Children’s Oncology Group
Source: PLoS One. 2016 Mar 18;11(3):e0151728. doi: 10.1371/journal.pone.0151728 (PMC4798297; doi:10.1371/journal.pone.0151728)
Supplement: S1 Fig — (PDF) [file pone.0151728.s002.pdf]

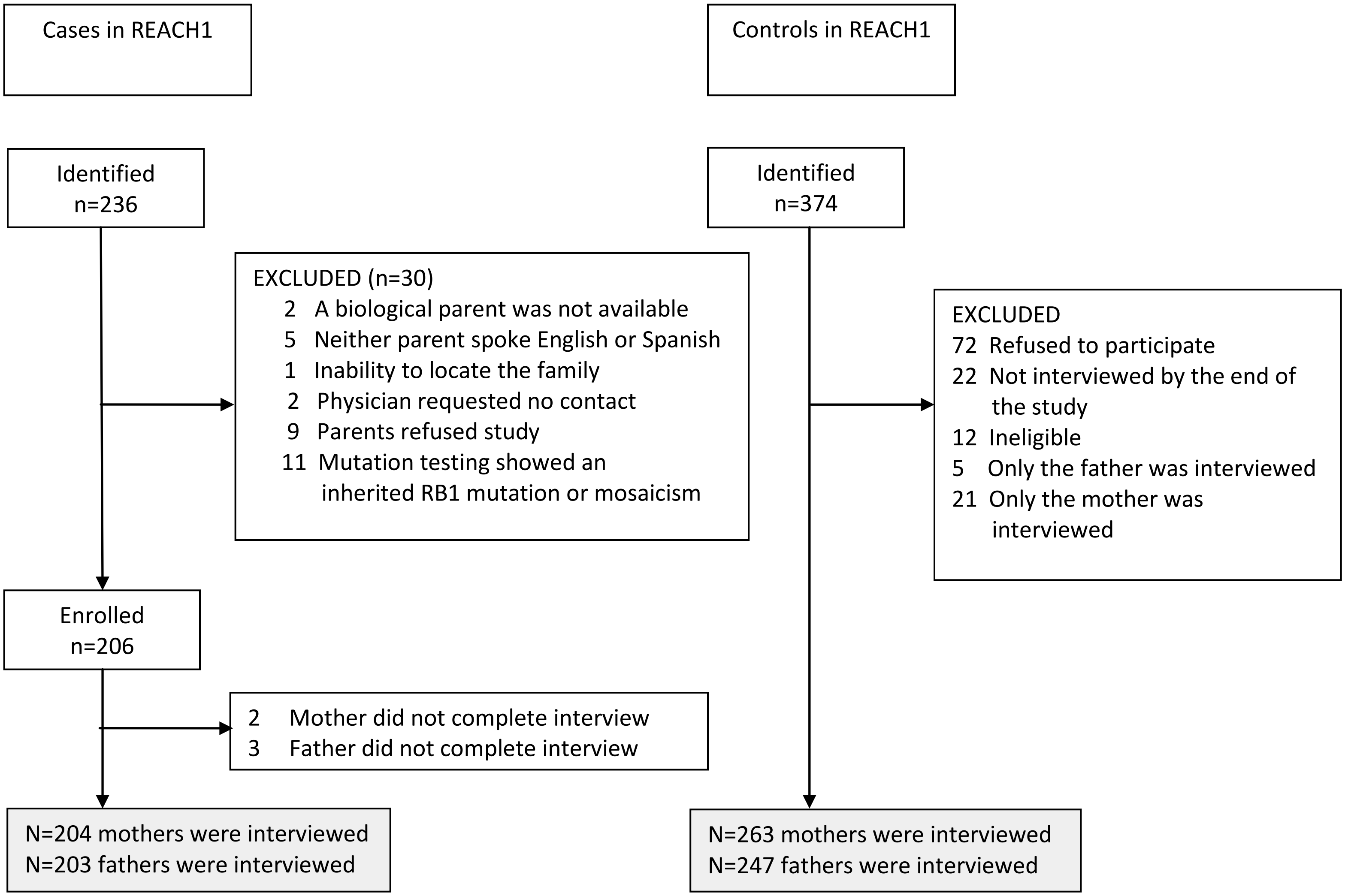

S Fig1. Recruitment of case and control parents for a study of sporadic bilateral retinoblastoma (REACH1 study)
