# Supplementary material for: Sporadic Retinoblastoma and Parental Smoking and Alcohol Consumption before and after Conception: A Report from the Children’s Oncology Group
Source: PLoS One. 2016 Mar 18;11(3):e0151728. doi: 10.1371/journal.pone.0151728 (PMC4798297; doi:10.1371/journal.pone.0151728)
Supplement: S2 Fig — (PDF) [file pone.0151728.s003.pdf]

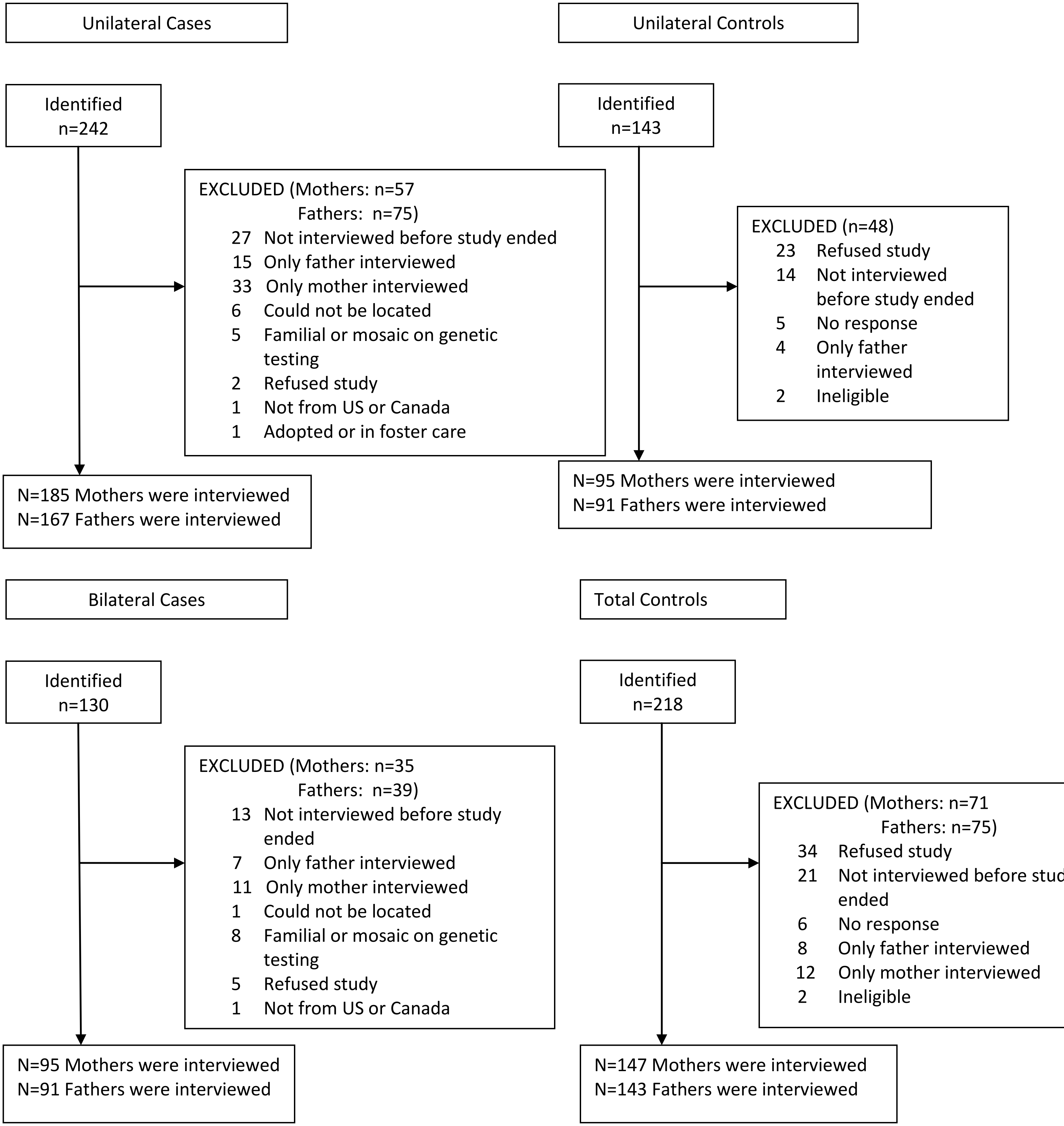
Unilateral Cases
Identified n=242
EXCLUDED (Mothers: n=57 Fathers: n=75)
27 Not interviewed before study ended
15 Only father interviewed
33 Only mother interviewed
6 Could not be located
5 Familial or mosaic on genetic testing
2 Refused study
1 Not from US or Canada
1 Adopted or in foster care
N=185 Mothers were interviewed
N=167 Fathers were interviewed
Unilateral Controls
Identified n=143
EXCLUDED (n=48)
23 Refused study
14 Not interviewed before study ended
5 No response
4 Only father interviewed
2 Ineligible
N=95 Mothers were interviewed
N=91 Fathers were interviewed
Bilateral Cases
Identified n=130
EXCLUDED (Mothers: n=35 Fathers: n=39)
13 Not interviewed before study ended
7 Only father interviewed
11 Only mother interviewed
1 Could not be located
8 Familial or mosaic on genetic testing
5 Refused study
1 Not from US or Canada
N=95 Mothers were interviewed
N=91 Fathers were interviewed
Total Controls
Identified n=218
EXCLUDED (Mothers: n=71 Fathers: n=75)
34 Refused study
21 Not interviewed before stud ended
6 No response
8 Only father interviewed
12 Only mother interviewed
2 Ineligible
N=147 Mothers were interviewed
N=143 Fathers were interviewed

S Fig2. Recruitment of case and control parents for a study of sporadic bilateral and unilateral retinoblastoma (REACH2 study)
