# Supplementary material for: Sporadic Retinoblastoma and Parental Smoking and Alcohol Consumption before and after Conception: A Report from the Children’s Oncology Group
Source: PLoS One. 2016 Mar 18;11(3):e0151728. doi: 10.1371/journal.pone.0151728 (PMC4798297; doi:10.1371/journal.pone.0151728)
Supplement: S1 Table — (PDF) [file pone.0151728.s004.pdf]

Table S1. Demographic characteristics of fathers in unmatched analyses of bilateral cases.

Characteristics	Unmatched [a] Controls (N=390) N (%)	Unmatched [a] Bilateral Cases (N=294) N (%)
**Father's race**		
White non-Hispanic	306 (78.7)	187 (63.6)
African American non-Hispanic	25 (6.4)	37 (12.6)
Hispanic	40 (10.3)	42 (14.3)
Other	18 (4.6)	28 (9.5)
Missing	1	0
**Father's educational attainment**		
<High school	20 (5.1)	30 (10.2)
High school graduate	65 (16.7)	76 (25.9)
Some college or other training	75 (19.2)	64 (21.8)
College graduate or more	230 (59.0)	123 (42.0)
Missing	0	1
**Father's age at child's birth**		
<25	33 (8.6)	31 (10.7)
25-29	78 (20.3)	57 (20.0)
30-34	141 (36.7)	87 (30.0)
35-39	89 (23.2)	81 (27.9)
40+	43 (11.2)	34 (11.7)
Missing	6	4
**Father's total household income**		
Less than $ 35,000	61 (17.1)	69 (26.0)
35,000- 50,000	54 (15.2)	45 (17.0)
50,000-75,000	84 (23.6)	58 (21.9)
More than $ 75,000	149 (41.9)	87 (32.8)
Refused	5 (1.4)	2 (0.8)
Do not know	3 (0.8)	4 (1.5)
Missing	34	29

[a] 390 fathers of 424 controls and 294 fathers of 301 bilateral cases were interviewed.
