# Supplementary material for: Sporadic Retinoblastoma and Parental Smoking and Alcohol Consumption before and after Conception: A Report from the Children’s Oncology Group
Source: PLoS One. 2016 Mar 18;11(3):e0151728. doi: 10.1371/journal.pone.0151728 (PMC4798297; doi:10.1371/journal.pone.0151728)
Supplement: S2 Table — (PDF) [file pone.0151728.s005.pdf]

Table S2. Demographic characteristics of mothers in unmatched analyses of unilateral cases

Characteristics	Unmatched [a] Controls (N=409) N (%)	Unmatched [a] Unilateral Cases (N=185) N (%)
**Mother's race**		
White non-Hispanic	320 (78.2)	105 (56.8)
African American non-Hispanic	25 (6.1)	14 (7.6)
Hispanic	41 (10.0)	46 (24.9)
Other	23 (5.6)	20 (10.8)
Missing	0	0
**Mother's educational attainment**		
<High school	14 (3.4)	15 (8.1)
High school graduate	52 (12.7)	32 (17.3)
Some college or other training	92 (22.5)	40 (21.6)
College graduate or more	251 (61.4)	98 (53.0)
Missing	0	0
**Mother's age at child's birth**		
<25	60 (14.7)	31 (16.8)
25-29	104 (25.4)	59 (31.9)
30-34	161 (39.4)	61 (33.0)
35-39	67 (16.4)	26 (14.1)
40+	17 (4.2)	8 (4.3)
Missing	0	0
**Total household income**		
Less than $35,000	81 (21.5)	56 (30.3)
$35,000- 50,000	67 (17.8)	18 (9.7)
$50,000-75,000	76 (20.2)	30 (16.2)
More than $75,000	145 (38.5)	62 (33.5)
Refused	3 (0.8)	8 (4.3)
Do not know	5 (1.3)	11 (6.0)
Missing	32	0

[a] 409 mothers of 424 controls and 185 mothers of 187 unilateral cases were interviewed.
