# Supplementary material for: Sporadic Retinoblastoma and Parental Smoking and Alcohol Consumption before and after Conception: A Report from the Children’s Oncology Group
Source: PLoS One. 2016 Mar 18;11(3):e0151728. doi: 10.1371/journal.pone.0151728 (PMC4798297; doi:10.1371/journal.pone.0151728)
Supplement: S3 Table — (PDF) [file pone.0151728.s006.pdf]

Table S3. Paternal smoking and alcohol consumption and bilateral retinoblastoma (unconditional logistic regression)

	Controls (N=390) N (%)	Bilateral cases (N=294) N (%)	Unconditional OR[a]	Adjusted OR (95 % CI)[b]
**Father ever smoked, lifetime**				
No	248 (63.6)	163 (55.1)	1.0	
Yes	142 (36.4)	132 (44.9)	1.5	1.1 (0.8, 1.7)
Missing	0	0		
**Father's lifetime smoking (pack-years)**				
0	248 (66.9)	162 (59.6)	1.0	
>0 to 5	62 (16.7)	38 (14.0)	1.0	0.9 (0.5, 1.5)
>5 to 10	33 (8.9)	31 (11.4)	1.4	1.1 (0.6, 2.1)
>10	28 (7.6)	41 (15.1)	2.8	1.9 (1.0, 3.8)
Missing	19	22		
**Father smoked in the year before pregnancy**				
Never smoked, lifetime	248 (63.6)	162 (55.1)	1.0	
Ever smoker, did not smoke in year before pregnancy	56 (14.4)	32 (10.9)	0.8	1.0 (0.6, 1.7)
Smoked in year before pregnancy	86 (22.1)	100 (34.0)	2.0	1.2 (0.8, 2.0)
Missing	0	0		
**Father's number of cigarettes per day, year before pregnancy**				
0	304 (78.8)	194 (66.9)	1.0	
1-9	33 (8.6)	32 (11.0)	1.7	1.0 (0.5, 1.9)
10+	49 (12.7)	64 (22.1)	2.3	1.7(0.9, 3.1)
Missing	4	4		
**Father drinking alcohol, year before pregnancy**				
0 drink	71 (18.2)	55 (18.8)	1.0	
<1 drink per week	73 (18.7)	49 (16.7)	1	1.0 (0.5, 1.8)
1-7 drinks per week	165 (42.3)	119 (40.6)	0.9	0.9 (0.5, 1.6)
1+ drinks per day	81 (20.8)	70 (23.9)	1.4	1.4 (0.8, 2.7)
Missing	0	1		
**Father's drinking ≥ 6 drinks per occasion in the year before pregnancy (binge drinking)**				
No	240 (61.7)	172 (59.1)	1.0	
Yes	149 (38.3)	119 (40.9)	1.1	1.0 (0.7, 1.5)
Missing	1	3		
**Father's drinking ≥ 6 drinks per occasion in the year before pregnancy (binge drinking)**				
Never	240 (61.7)	172 (59.1)	1.0	
< Once per month	67 (17.2)	71 (24.4)	1.4	1.5 (0.9, 2.4)
Monthly	45 (11.6)	24 (8.3)	0.7	0.6 (0.3, 1.2)
Weekly	27 (6.9)	19 (6.5)	0.9	0.7 (0.3, 1.4)
Daily or almost daily	10 (2.6)	5 (1.7)	1.0	0.9 (0.3, 3.3)
Missing	1	3		

[a] Crude OR in unconditional logistic regression model is adjusted for matching variable, age.

[b] Smoking and drinking analyses adjusted for the matching variable (child age at interview), father's race, father's educational attainment, father's household income, father's age at child's birth. In addition, smoking analyses adjusted for father's drinking in the year before pregnancy, and the mother's smoking. Drinking analysis adjusted for father’s smoking in the year before pregnancy, and the mother's drinking.
