# Supplementary material for: Sporadic Retinoblastoma and Parental Smoking and Alcohol Consumption before and after Conception: A Report from the Children’s Oncology Group
Source: PLoS One. 2016 Mar 18;11(3):e0151728. doi: 10.1371/journal.pone.0151728 (PMC4798297; doi:10.1371/journal.pone.0151728)
Supplement: S4 Table — (PDF) [file pone.0151728.s007.pdf]

Table S4. Maternal smoking and drinking and unilateral retinoblastoma (unconditional logistic regression analysis)

	Controls (N=409) N (%)	Unilateral cases (N=185) N (%)	Unconditional OR [a]	Adjusted OR (95 % CI) [b]
**Mother ever smoked, lifetime**				
No	277 (67.7)	108 (58.4	1.0	
Yes	132 (32.3)	77 (41.6)	1.6	1.7 (1.0, 2.8)
Missing	0	0		
**Mother smoked in the year before pregnancy**				
Never smoked, lifetime	277 (67.7)	108 (58.4)	1.0	
Ever smoker,did not smoke in year before	71 (17.4)	30 (16.2)	1.1	1.3 (0.7, 2.3)
Smoked in year before pregnancy	61 (14.9)	47 (25.4)	2.2	2.4 (1.3. 4.7)
Missing	0	0		
**Mother's cigarettes per day, year before pregnancy**				
0	348 (85.7)	138 (75.8)	1.0	
1-9	24 (5.9)	26 (14.3)	3.2	2.7 (1.2, 6.0)
10+	34 (8.4)	18 (9.9)	1.5	1.7 (0.7, 3.9)
Missing	3	3		
**Mother drinking alcohol, year before pregnancy**				
0	125 (30.8)	41 (22.2)	1.0	
<1 drink per week	122 (30.1)	73 (39.5)	1.8	2.0 (1.1, 3.6)
1-7 drinks per week	133 (32.8)	58 (31.4)	1.3	1.3 (0.7, 2.6)
1+ drinks per day	26 (6.4)	13 (7.0)	1.5	1.1 (0.4, 3.0)
Missing	3	0		
**Mother's drinking ≥ 6 drinks per occasion in the year before pregnancy (binge drinking)**				
No	341 (83.4)	137 (74.1)	1.0	
Yes	68 (16.6)	48 (25.9)	1.7	1.5 (0.8, 2.5)
Missing	0	0		

[a] Crude OR in unconditional logistic regression model is adjusted for matching variable, age.

[b] Smoking and drinking analyses both adjusted for the matching variable (child age at interview), mother's race, mother's educational attainment, household income, and the mother's age at child's birth. In addition, smoking analyses are adjusted for mother's drinking in the year before pregnancy, and mutually the father's smoking; and drinking variables adjusted for the mother's smoking in a year before pregnancy, and mutually the father's drinking.

Table S4 (continued). Maternal smoking and drinking and unilateral retinoblastoma (unconditional logistic regression analysis)

	Controls (N=144) N (%)	Unilateral cases (N=185) N (%)	Unconditional OR[a]	Adjusted OR (95 % CI)[b]
**Mother's smoking in the month before or during pregnancy**				
Never smoked, lifetime	101 (70.1)	107 (58.2)	1.0	
No	26 (17.1)	35 (19.0)	1.3	1.7 (0.8, 3.3)
Yes	17 (11.8)	42 (22.8)	2.5	2.8 (1.1, 7.0)
Missing	0	1		
**Mother's smoking in the first trimester**				
No	136 (94.4)	159 (86.4)	1.0	
Yes	8 (5.6)	25 (13.6)	2.8	3.7 (1.2, 11.6)
Missing	0	1		
**Mother's smoking in the second trimester**				
No	138 (95.8)	169 (91.9)	1.0	
Yes	6 (4.2)	15 (8.2)	2.0	1.7 (0.5, 6.5)
Missing	0	1		
**Mother's smoking in the third trimester**				
No	138 (95.8)	168 (91.3)	1.0	
Yes	6 (4.2)	16 (8.7)	2.1	1.9 (0.5, 7.1)
Missing	0	1		
**Mother's drinking in the month before pregnancy**				
No	70 (48.6)	84 (46.2)	1.0	
Yes	74 (51.4)	98 (53.9)	1.1	1.3 (0.7, 2.4)
Missing	0	3		
**Mother's drinking in the first trimester**				
No	130 (90.3)	165 (89.7)	1.0	
Yes	14 (9.7)	19 (10.3)	1.0	1.1 (0.4, 2.7)
Missing	0	1		
**Mother's drinking in the second trimester**				
No	135 (93.8)	177 (96.2)	1.0	
Yes	9 (6.3)	7 (3.8)	0.6	1.1 (0.3, 3.9)
Missing	0	1		
**Mother's drinking in the third trimester**				
No	136 (94.4)	177 (96.2)	1.0	
Yes	8 (5.6)	7 (3.8)	0.7	0.7 (0.2, 2.8)
Missing	0	1		

[a] OR in unconditional logistic regression model is adjusted for matching variable, age.

[b] Smoking variables and drinking variables both adjusted for matching variable (child age at interview), mother's race, mother's educational attainment, household income, and the mother's age at child's birth. In addition, smoking analyses are adjusted for the mother's drinking in the year before pregnancy, and mutually the father's smoking variable; drinking analyses adjusted for the mother’s smoking in the year before pregnancy, and mutually the father's drinking.
