# Supplementary material for: Sporadic Retinoblastoma and Parental Smoking and Alcohol Consumption before and after Conception: A Report from the Children’s Oncology Group
Source: PLoS One. 2016 Mar 18;11(3):e0151728. doi: 10.1371/journal.pone.0151728 (PMC4798297; doi:10.1371/journal.pone.0151728)
Supplement: S5 Table — (PDF) [file pone.0151728.s008.pdf]

Table S5. Environmental tobacco exposure of the mother during pregnancy and unilateral retinoblastoma (unconditional logistic regression)

	Controls	Unilateral cases	Unconditional	
			Crude	Adjusted
	N (%)	N (%)	OR [a]	OR (95% CI)[b]
Mother never smoked, father and mother lived together [c]				
**Father ever smoked, lifetime**				
No	184 (75.7)	56 (63.6)	1.0	
Yes	59 (24.3)	32 (36.4)	1.8	1.9 (1.0, 3.6)
Missing	0	0		
**Father's life time smoking (pack-years)**				
0	184 (77.3)	56 (67.5)	1.0	
>0 to 5	34 (14.3)	20 (24.1)	2.0	1.9 (0.9, 4.0)
>5 to 10	14 (5.9)	5 (4.8)	1.0	1.6 (0.3, 7.4)
>10	6 (2.5)	4 (3.6)	1.8	1.3 (0.2, 7.6)
Missing	5	3		
**Father smoked in the year before pregnancy**				
Never smoked, life time	184 (75.7)	56 (63.6)	1.0	
Ever smoker but did not smoke in the year before	32 (13.2)	14 (15.9)	1.4	1.4(0.6, 3.3)
Smoked in the year before pregnancy	27 (11.1)	18 (20.5)	2.3	2.5 (1.1, 6.0)
Missing	0	0		
Mother and father lived together [d]				
Both father and mother are never smokers, lifetime	184 (51.8)	56 (37.8)	1.0	
Father ever smoker and mother never smoker, lifetime	59 (16.6)	32 (21.6)	1.8	1.8 (1.0, 3.4)
Father never smoker and mother ever smoker, lifetime	45 (9.0)	28 (18.9)	2.2	2.7 (1.4, 5.2)
Both father and mother ever smoked, lifetime	67 (13.3)	32 (21.6)	1.7	2.2 (1.2, 4.2)
Missing	0	0		

[a] OR in unconditional logistic regression model adjusted for the matching variable, age.
[b] Adjusted for the matching variable (child's age at interview), mother's race, mother's educational attainment, household income, mother's age at child's birth, and the mother’s alcohol drinking in the year before pregnancy.
[c] 243 controls and 88 unilateral cases.
[d] 355 controls and 148 unilateral cases.
