# Supplementary material for: Sporadic Retinoblastoma and Parental Smoking and Alcohol Consumption before and after Conception: A Report from the Children’s Oncology Group
Source: PLoS One. 2016 Mar 18;11(3):e0151728. doi: 10.1371/journal.pone.0151728 (PMC4798297; doi:10.1371/journal.pone.0151728)
Supplement: S6 Table — (PDF) [file pone.0151728.s009.pdf]

Table S6. Maternal smoking and drinking consumption and unilateral retinoblastoma (Conditional logistic regression)

	Controls (N=95) N (%)	Unilateral cases (N=95) N (%)	OR	Conditional Adjusted OR (95 % CI)[a]
**Mother's smoking in the second trimester**				
No	91(97.8)	88 (93.6)	1.0	
Yes	2 (2.2)	6 (6.4)	NE	NE
Missing	2	1		
**Mother's smoking in the third trimester**				
No	91 (97.8)	88 (93.6)	1.0	
Yes	2 (2.2)	6 (6.4)	NE	NE
Missing	2	1		
**Mother's drinking in the month before pregnancy**				
No	45 (48.4)	39 (41.9)	1.0	
Yes	48 (51.6)	54 (58.1)	1.3	1.2 (0.4, 3.5)
Missing	2	2		
**Mother's drinking in the first trimester**				
No	84 (90.3)	85 (90.4)	1.0	
Yes	9 (9.7)	9 (9.6)	1.0	0.8 (0.2, 3.1)
Missing	2	1		
**Mother's drinking in the second trimester**				
No	86 (92.5)	91 (96.8)	1.0	
Yes	7 (7.5)	3 (3.2)	0.3	0.3 (0.0, 3.8)
Missing	2	1		
**Mother's drinking in the third trimester**				
No	87 (93.6)	91 (96.8)	1.0	
Yes	6 (6.4)	3 (3.2)	0.4	0.4 (0.0, 3.4)
Missing	2	1		

[a] Smoking and drinking analyses adjusted for mother's race, mother's educational attainment, household income, mother's age at child's birth. In addition, smoking analyses adjusted for mother's drinking in the year before pregnancy, and mutually father's smoking; and drinking analyses adjusted for mother's smoking in the year before pregnancy, and mutually father's drinking.

NE=Not Estimable.
